# Using the Recommended Summary Plan for Emergency Care and Treatment (ReSPECT) in a community setting: does it facilitate best interests decision-making?

**DOI:** 10.1136/jme-2024-110144

**Published:** 2025-01-19

**Authors:** Karin Eli, Celia J Bernstein, Jenny Harlock, Caroline J Huxley, Julia Walsh, Hazel Blanchard, Claire A Hawkes, Gavin D Perkins, Chris Turner, Frances Griffiths, Anne-Marie Slowther

**Affiliations:** 1 Warwick Medical School, University of Warwick, Coventry, UK; 2 Forrest Medical Centre, Coventry, UK; 3 King's College London Florence Nightingale Faculty of Nursing Midwifery & Palliative Care, London, UK; 4 University Hospitals Coventry and Warwickshire NHS Trust, Coventry, UK

**Keywords:** Decision Making, Ethics- Medical, Palliative Care

## Abstract

In the UK, the Recommended Summary Plan for Emergency Care and Treatment (ReSPECT) is a widely used process, designed to facilitate shared decision-making between a clinician and a patient or, if the patient lacks capacity to participate in the conversation, a person close to the patient. A key outcome of the ReSPECT process is a set of recommendations, recorded on the patient-held ReSPECT form, that reflect the conversation. In an emergency, these recommendations are intended to inform clinical decision-making, and thereby enable the attending clinician—usually a general practitioner (GP) or paramedic—to act in the patient’s best interests. This study is the first to explore the extent to which ReSPECT recommendations realise their goal of informing best interests decision-making in community contexts. Using a modified framework analysis approach, we triangulate interviews with patients and their relatives, GPs and nurses and care home staff. Our findings show that inconsistent practices around recording patient wishes, diverging interpretations of the meaning and authority of recommendations and different situational contexts may affect the interpretation and enactment of ReSPECT recommendations. Enacting ReSPECT recommendations in an emergency can be fraught with complexity, particularly when attending clinicians need to interpret recommendations that did not anticipate the current emergency. This may lead to decision-making that compromises the patient’s best interests. We suggest that recording patients’ values and preferences in greater detail on ReSPECT forms may help overcome this challenge, in providing attending clinicians with richer contextual information through which to interpret treatment recommendations.

## Introduction

In the UK, healthcare professionals have a legal obligation to make decisions about a person’s medical treatment and care when that person lacks capacity, and where there is no proxy decision-maker. These decision-makers are legally and morally obligated to act in the person’s best interests.^1 2^ Deciding what is in someone’s best interests can be challenging, and includes taking into account their medical needs, range of treatment options, the likely benefits and burdens of treatment and what the person’s views and wishes regarding treatment might be if they were able to articulate them. While there is considerable academic debate about the meaning of best interests, the predominant position in many healthcare systems prioritises or emphasises the importance of the patient’s wishes and feelings, and thus patient autonomy, in best interests decision-making. Recent commentaries on UK Court of Protection decisions suggest an increasing focus on prioritising the patient’s wishes and feelings in determining best interests.^3 4^

In an emergency, or when someone’s clinical condition is rapidly deteriorating, decisions often need to be made with limited information, particularly regarding the person’s wishes and preferences. The development of emergency care treatment plans (ECTPs) reflects attempts to address this challenge by recording specific treatment recommendations for future anticipated clinical scenarios informed by prior discussion with the patient (or someone close to the patient if the patient lacks capacity). In the USA, a model of ECTP, Physician Orders for Life Sustaining Treatment (POLST), is now used in most states; however, there is some concern its widespread use may curtail person-centred decision-making, contrary to its intended aim.^5^

**Figure 1:**
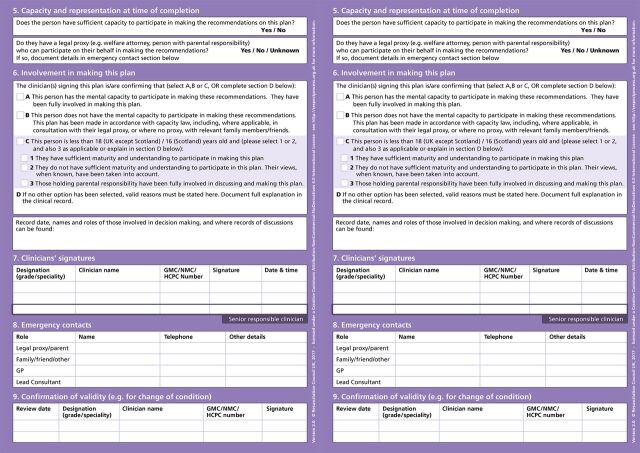
ReSPECT form, V.2.0. CPR, cardiopulmonary resuscitation; GP, general practitioner; NHS, National Health Service; ReSPECT, Recommended Summary Plan for Emergency Care and Treatment.

**Figure 2:**
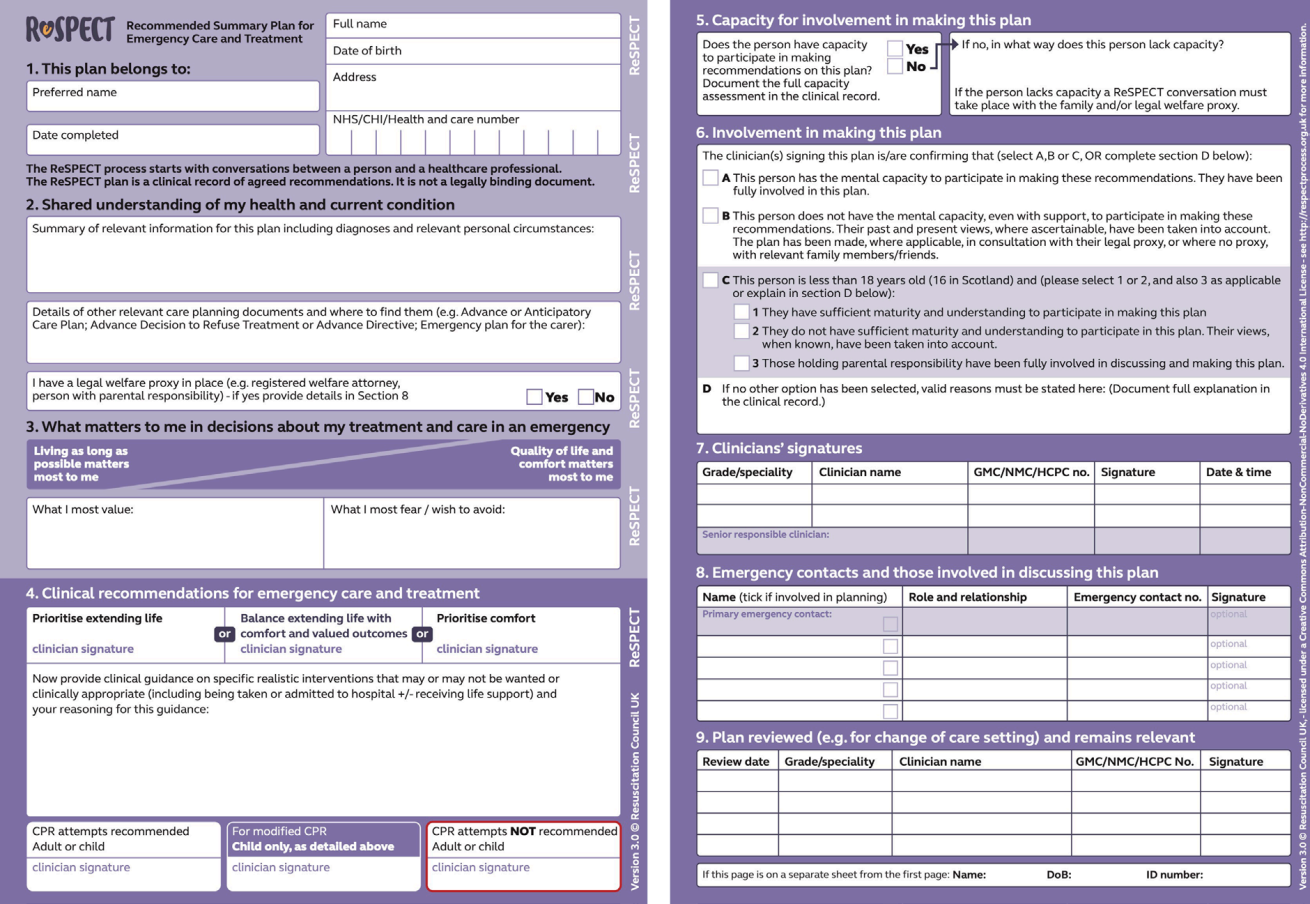
ReSPECT form, V.3.0. CPR, cardiopulmonary resuscitation; NHS, National Health Service; ReSPECT, Recommended Summary Plan for Emergency Care and Treatment.

In the UK, The Recommended Summary Plan for Emergency Care and Treatment (ReSPECT) is a widely used ECTP, launched in 2016 by the Resuscitation Council UK. ReSPECT is conceptualised as a shared decision-making process, which emphasises a conversation between a clinician (usually a doctor or a senior nurse) and a patient (or, if the patient lacks capacity, someone close to them), to explore treatment options that reflect what is clinically possible and aligns with the patient’s values.^6^ The clinician records conversation outcomes on the ReSPECT form with specific free-text recommendations about future treatment, including cardiopulmonary resuscitation (CPR).^7^ The ReSPECT form is designed to be patient-held and transition with the patient across health and care settings (see figures 1 and 2).^6^

As part of a multicentre study to investigate ReSPECT use in community settings, we interviewed primary care clinicians, care home staff, patients and relatives about their experiences of ReSPECT.^8^ We report our analysis of their experiences elsewhere.^9 10^ In this paper, we consider the impact of ReSPECT on best interests decision-making in emergencies. Our analysis is framed within a concept of best interests that emphasises the wishes, feelings and values of the patient for whom the decision is being made, reflecting the current position of UK law. Whether patient wishes and feelings determine best interests or contribute to a more multifaceted approach, sound knowledge of the patient’s wishes and feelings will facilitate the decision-making process.

## Methods

### Study design

We conducted a qualitative analysis of interviews with key stakeholders, using a normative framework of best interests.

### Participant recruitment

Thirteen general practices in three geographical areas in England participated in the study. We invited general practitioners (GPs) and specialist nurses involved in ReSPECT conversations to participate. Each practice identified patients with a ReSPECT plan completed in the previous six months and posted an invitation letter and information sheet to eligible patients or, if the patient lacked capacity, their next of kin; patients who were currently in hospital or were imminently approaching the end of life were not contacted. Participating practices identified care homes where they had registered patients and sent them information about the study. In care homes that expressed an interest, staff were recruited through purposive sampling for seniority and involvement with ReSPECT. Additionally, we advertised the study to care homes not linked to the participating practices through local research networks.

### Data collection

We developed topic guides for each interview type. Clinician topic guides captured accounts of recent cases in which participants completed a ReSPECT plan for a patient, using these to ground a more general discussion of their experiences of using ReSPECT. Patient and relative topic guides captured their narratives of ReSPECT, from the pre-conversation stage to the present day, and their reflections on the process. Care home staff topic guides captured their understandings, experiences and challenges related to ReSPECT.

Semi-structured interviews were conducted on Microsoft Teams (clinicians and care home staff members), via telephone (clinicians, patients and relatives) or in person (all groups) (table 1). Four researchers (CJB, JH, KE and JW) conducted the clinician interviews, three (JH, KE and CJH) the patient and relative interviews and two (JH and CJB) the care home staff interviews. All interviews were recorded and professionally transcribed, except for one clinician interview and one group interview with five care home staff in which the participants requested that written notes be taken instead.

**Table 1:** Sample

Interview type	Number of interviews	Participants
Patient/relative	13 (nine via telephone; four in person, at the participant’s home)	16 (six patients, four relatives and three patient and relative pairs)
Care home staff members	23 (12 in person, at the participant’s workplace; 11 on Microsoft Teams)	31 (16 care coordinators/senior carers, 12 care home managers/deputy managers, three senior nurses/nursing leads)
Primary care clinicians	26 (25 on Microsoft Teams; one in person, at the participant’s workplace)	26 (21 GPs and five specialist nurses)
**Total**	**62**	**73**

GP, general practitioner.

Before the interviews, all participants provided informed consent, either verbally (online and telephone interviews) or in writing (in-person interviews). This manuscript uses pseudonyms for all participants, and all identifying details have been omitted.

### Analysis

The analysis included two stages. In the first stage, we used a modified framework analysis approach,^11^ guided by an ethical lens, whereby we looked for explicit and implicit ethical issues and concepts within the data. Twelve transcripts (five patient/relative, four clinician and three care home staff interviews) were thematically analysed by six members of the study team (KE, CJB, JH, CJH, FG and A-MS): four social scientists, one GP/medical sociologist and one GP/medical ethicist; 11 of these transcripts were coded by two or more study team members. While focused on identifying ethical issues, this initial analysis used an open coding approach.^12^ Following team discussion, a draft coding framework was developed by A-MS.

Interview transcripts were then summarised by KE and A-MS, following a pen portrait approach.^13^ To achieve code saturation, 37 individual pen portraits were created, including all patient/relative interviews and half of the clinician and care home staff interviews. Using the draft coding framework, KE and A-MS undertook codebook thematic analysis, in line with the framework analysis approach,^11^ with KE coding all pen portraits and A-MS coding 40%; the process allowed for the refining of existing codes and categories and for the development of new ones.^12^ Intercoding was compared in meetings which found analysis highly consistent. The remaining interview transcripts were then reviewed by A-MS; this process yielded no additional codes, suggesting that meaning saturation had been achieved.^14^ This analysis led to a framework with eight categories (see online supplemental material). We have reported on the results of this initial descriptive analysis in the overall study report.^15^

10.1136/jme-2024-110144.supp1 Supplementary data

In the second stage of analysis, on which we report in the current paper, we deductively analysed the data across these categories to explore to what extent the ReSPECT process facilitated best interests decision-making in emergency situations, guided by the following questions:

- To what extent do ReSPECT plans reflect patient wishes/preferences?
- How do ReSPECT recommendations inform best interests decision-making (defined as balancing harms and benefits of treatment, taking into account patient preferences and values)?
- What external constraints impact on implementation of patient wishes as recorded in ReSPECT recommendations?

These questions reflect our normative positioning on best interests as emphasising patient wishes, preferences and values. The extracted data across all categories in our primary analysis were deductively analysed using our established codes and recategorised into three topics that mapped to our three questions around best interests (table 2).^16^ Thus, the current paper focuses on the best interests thread running through these identified categories, adding a layer of interpretation to the original framework.

**Table 2:** Categories and codes for deductive analysis within a best interests normative framework

Topic	Codes
ReSPECT plans vary in the extent to which they reflect individual autonomy	Control of ReSPECT initiation Changing priorities during illness course Patients’ views not considered (in completion or in use of form) Control of form/information Patient understanding Patient capacity Empowers patient/reflects patient wishes Advocating for one’s own wishes Timing of conversation Cannot address all scenarios
Interpretation of ReSPECT recommendations in an emergency is complex and variable	Assumptions that form is DNACPR (do not attempt cardiopulmonary resuscitation) Recommendations not being followed Form protects patient from inappropriate treatment Difficulty interpreting form Form inadequately completed Form prevents patient from receiving treatment Responding to changes in healthcare needs Advocating for the patient Concern that recommendations will not be followed Confidence that recommendations will be followed Family’s wishes/interests Clinician responsibility to make a best interests decision
Healthcare system constraints may impact on the implementation of ReSPECT recommendations	Reduces admissions Strain on staff resources Recommendations not feasible in current situation

## Findings

Sixty-two interviews with 73 participants took place; for sample characteristics see table 1. In what follows, we describe the themes (topics) identified in our deductive analysis, framed by our three questions (table 2).

### ReSPECT plans vary in the extent to which they reflect individual autonomy

Patient participants expressed confidence that the recommendations recorded on their ReSPECT plans would be followed in an emergency, thereby protecting them from receiving treatment they would not want—in most cases, CPR. As one participant said:

your own wishes are in black and white for, for someone to read. And, and that’s there, so they have to follow what you’ve put down. It’s not just saying to one GP what you, what you want, and that GP happens to be not around at that particular time. It’s all here and, and signed off by a doctor. (Patient 12)

Although patient participants varied in the extent to which they remembered the ReSPECT conversation, all said the recorded recommendations reflected their wishes. However, not all patient or relative participants recalled a conversation about what they valued in terms of medical treatment other than CPR, which was sometimes the only recommendation recorded on the form. One participant described to the researcher what she would have wanted the form to include in response to questions like ‘what is important to you?’, saying these questions had been left blank on her form.

Care home staff, GPs and nurses spoke of exploring patients’ wishes as central to the ReSPECT process. For example, when describing a ReSPECT conversation with a terminally ill patient, one nurse said:

So we discussed his future wishes, what’s important to him, what’s important to him in terms of treatment going forward or not (…) and then formulating that into a ReSPECT Form and into a, into a care plan. (Specialist nurse 5, Area 3)

Nonetheless, GPs and nurses also described challenges in holding ReSPECT conversations when patients found it difficult to imagine or understand future clinical scenarios and treatment options, meaning their preferences could not be translated easily into recommendations.

When GPs and nurses spoke about exploring what was important to patients, they often maintained a clinical focus, with the discussion centred on treatment. A few patient participants, however, expressed a more nuanced understandings of the relationships between values and ReSPECT recommendations. For example, one participant said:

…if I could be brought back to consciousness and given a week or two of consciousness to resolve any outstanding financial matters, then I might take that. If the best it could do is just keep me alive in a non-sentient state where I wouldn’t be able to contribute any further to these questions about my financial future, then I probably wouldn’t want it. (Patient 3)

ReSPECT recommendations reflected the patient’s current condition and preferences. However, clinician participants said ReSPECT plans were not reviewed regularly, mainly due to time constraints. Instead, they relied either on the patient asking for a review, or on their own assessment of deterioration in the patient’s condition. Relying on patients to trigger the review process could be problematic. We found in our interviews with patients and relatives that while some knew ReSPECT forms could be reviewed, others did not.

### Interpretation of ReSPECT recommendations in an emergency is complex and variable

In our sample, care home staff witnessed how ReSPECT recommendations were used in emergencies, and much of this section focuses on their perspectives. Care home staff reported that ReSPECT recommendations were often non-specific (using phrases such as ‘for ward-based care’ or ‘not for admission’) or did not reflect the current clinical situation. In practice, this could lead to diverging interpretations of ReSPECT recommendations by attending clinicians (usually paramedics or GPs) in an emergency, resulting in decisions that care home staff perceived as not in the patient’s best interests. For example, care home staff sometimes felt the attending clinician failed to consider that the ReSPECT recommendations did not capture the current situation, following them to the detriment of the patient’s care. As one participant explained:

…you have to push quite hard then to get them [residents] admitted into hospital. So, just because the form says that it’s not explicit for every circumstance. Circumstance changes, it’s every situation is different, isn’t it? And you can’t foresee everything on the ReSPECT form, and some people take it quite literally. But that’s not happened once or twice. That’s been quite frequent. (Care home manager/deputy manager 2)

In other situations, attending clinicians appeared to go against ReSPECT recommendations that other staff involved in the patient’s care thought were relevant. In one case, a nurse described a patient being admitted to the hospital, despite her ReSPECT form indicating she was not for admission:

[the patient] ended up being in hospital for a week without any real confirmed diagnosis… And speaking to her relatives, you know, they were just distraught. (Specialist nurse 3, Area 1)

In another example, a care home staff member described a patient not being admitted despite the recommendations appearing to indicate admission was relevant in this situation:

…we had somebody who was for admission for all reversible, for a reversible condition, so if they had a chest infection but they needed hospital treatment to reverse the symptoms or make them better. And they didn’t go to hospital. The ambulance crew on that occasion said, “The decision rests with us.” (Care home manager/deputy manager 5)

On occasion, attending clinicians conflated a ReSPECT recommendation that the patient not be admitted to the hospital with an assumption that the patient was for ‘end-of-life’ care or not for other treatment.

I’ve had three, about three months ago where the paramedics came, saw the ReSPECT Form and said, phone the relative and said, “She’s end of life,” but she wasn’t end of life, it was just because she had a ReSPECT Form saying, “Not for admission for hospital unless discussed with relatives.” (GP 6, Area 2)

And I think then, the problem I think that happens there is then they’re [paramedics] interpreting that maybe they can’t give treatment because it says, “Not for admission.” (Care home manager/deputy manager 5)

As these cases show, while ReSPECT recommendations were recorded with the intention to equip clinicians to act in the patients’ best interests, as they were open to interpretation, in some emergency situations this aim was not realised.

Clinicians attending a patient in an emergency, often out of hours, may not have detailed knowledge of the patient with which to interpret ReSPECT recommendations when making a decision in the patient’s best interests. As one GP participant explained:

…obviously if it’s not within [GP practice opening] hours, we have to be at the mercy of kind of paramedics and out-of-hours doctors or clinicians making that judgment call. And I think the ReSPECT form can only do so much in that it can give the background to the patient and the baseline roughly of what that patient’s usually like and what the kind of family are aware of and what the wishes of the family are, what the wishes they believe of the patient are, but it comes down to their clinical judgment I think at the end of the day as to whether they believe something is reversible and therefore hospital admission is warranted or not. (GP 20, Area 3)

As captured in this quote, the ReSPECT plan can, at best, provide information for the clinician who does not know the patient to assist in making a best interests decision. The degree to which the patient’s values and preferences are explicit in the ReSPECT plan, and the detail provided in the clinical recommendations, will ultimately affect how person-centred the best interests decision can be.

### Healthcare system constraints may impact on the implementation of ReSPECT recommendations

Even if patient preferences are clearly articulated in recommendations, external constraints may limit the extent to which they can be enacted. For example, a GP described how, where frail patients wished to be for active treatment, hospitals’ concerns over resources could compromise the implementation of patient wishes:

if patients are very frail, have an end-of-life condition but they would want all active treatment, you know you’re going to have a difficult conversation with the bed manager if you, if you ring up and want to admit them. You have to sort of justify why this patient should be admitted when they are in such a, a frail case, so yeah, I think that’s a difficulty. (GP 17, Area 1)

Another constraint was the availability of care at home. One GP described how, as a junior doctor working in hospital, they encountered patients who had been admitted despite their ReSPECT plan stating otherwise:

And it may have been that carers were just not happy to leave them, it may be that they didn’t have adequate medications or symptom control in place to enable them to stay at home. Maybe they just didn’t have the social care framework in place for, to enable them to stay at home or it may be that whoever has made that decision hasn’t seen the form. (GP 20, Area 3)

Similarly, a nurse described having to move end-of-life patients to a hospice despite their previously expressed wishes, due to inability to manage symptoms at home. Another nurse cautioned that home care-related constraints are not always taken into account— although they should be—when clinicians interpret ReSPECT recommendations:

One recently that I’ve had is a gentleman who’s got [condition]. Didn’t have any carers at home, so he wasn’t safe to be discharged, but it’s, “Well, you know, you’ve got a ReSPECT form. You shouldn’t be here.” But, actually, there’s other factors, as well, that are going on, if that makes sense. (Specialist nurse 1, Area 2)

## Discussion

In this study of ReSPECT use in primary care, we found that inconsistent practices around recording patient wishes, diverging interpretations of the meaning and authority of recommendations and different situational contexts affect how ReSPECT plans impact on best interests decision-making in an emergency. While all participants—GPs and specialist nurses, care home staff and patients and relatives—emphasised the importance of recording patients’ wishes, preferences and values, in practice, plans did not always reflect this. Additionally, while both clinicians and care home staff described ReSPECT conversations as person-centred, in an emergency, many said that ReSPECT recommendations were not always followed and some expressed concern that variation in the interpretation of recommendations could lead to suboptimal treatment for some patients.

Our findings suggest the use of ReSPECT as currently practiced in community settings needs to be viewed with caution when informing best interests decisions regarding patient treatment in an emergency. There is substantial variation in the extent to which ReSPECT recommendations record or reflect patient wishes, preferences or values sufficiently to fulfil the requirement of best interests decision-making under the Mental Capacity Act.^1^ We have identified similar uncertainties in ReSPECT plan completion in a previous study conducted in UK hospitals, where time constraints and concerns about protecting patients from harm led to inconsistencies in the degree to which patient preferences and values were explored.^17–20^ The lack of a structured framework for ReSPECT plan review creates uncertainty about the relevance of recorded preferences in emergencies. As Zivkovic argues, anticipatory plans present patients with a linear vision of the future that cannot predict the dynamics of illness, care and sociality.^21^ These concerns about the validity of patient preferences recorded in ECTPs have also been noted in relation to POLST.^5 22 23^

Moreover, as with other anticipatory decision-making models,^23–25^ ReSPECT recommendations cannot anticipate every possible emergency scenario, meaning that recommendations must be subject to interpretation during an emergency. For some clinicians, ReSPECT recommendations contribute to the best interests decision-making process but do not have to be followed, while others view ReSPECT recommendations as determinative within the best interests assessment. This can lead to conflict between carers and clinicians, and potential harm to patients. Additionally, our finding that patient and relative participants expect ReSPECT recommendations will be followed in all situations demonstrates a gap between patient/relative expectations and clinical practice. Clinicians should make patients and relatives aware that ReSPECT guides, but does not prescribe, clinical decision-making.

The uncertainty and inconsistency in the interpretation of ReSPECT recommendations identified in our study reflects diverging concepts of best interests as synonymous with, informed by or distinct from preferences.^26^ Broadly speaking, in clinical practice, best interests is conceptualised as reflecting and informed by the patient’s values/wishes/preferences, but not determined exclusively by patient preferences.^27^ Recent cases in the UK Court of Protection suggest increasing prioritisation of the person's wishes and preferences in best interests decisions, giving greater weight to individual autonomy.^4^ However, understanding previously expressed wishes for particular treatments in specific emergency situations is challenging, given the uncertainty of many illness trajectories. To respect the patient’s autonomy, an understanding of who they are as a person and what is important to them may be more meaningful.

In theory, ECTPs, including ReSPECT, can facilitate best interests decision-making in an emergency by providing evidence of the patient’s preferences regarding treatment and an indication of what is important to them for an acceptable life. However, the patient’s preferences may have changed over time, and it is sometimes unclear whether patient preferences and values were sufficiently explored during the ReSPECT conversation and considered when writing recommendations.

This ambiguity regarding the weight to be given to ReSPECT recommendations can be challenging for clinician decision-makers and may lead to treatment decisions inconsistent with what the patient would have wanted or their overall best interests. As commentaries on POLST have noted, these risks are reduced when ECTPs are used for a person with a specific life-limiting illness and clearly defined future emergency scenarios.^5 22^ However, this is not the case for many people for whom ECTPs such as ReSPECT are being advocated. The importance of context and flexibility in using and interpreting tools such as ReSPECT for individual patients needs to be carefully considered. In the UK, previous experience with end-of-life care protocols, such as the Liverpool Care Pathway, underscores this message.^28^ This reflects a wider discourse on the tension between clinical guidelines and individualised patient care.^29^

Our findings convey the importance of including information about patients’ values and preferences on ReSPECT plans, beyond binary decision-making for or against certain treatments. In our earlier study of hospital-issued ReSPECT forms, we found that information on patient preferences was rarely recorded.^7^ In our recent analysis of community-issued ReSPECT forms, just over half of the forms featured information on patient preferences and/or values.^15 30^ Explicitly considering the person’s previously expressed values and preferences is part of a best interests decision-making process and is a requirement under the Mental Capacity Act.^1^ Recording such information reflects the values of person-centred care^31^ and, in an emergency, would allow clinicians to interpret relevant recommendations contextually and make best interests decisions aligned with patients’ values.

This study is the first to triangulate viewpoints from key stakeholders in the ReSPECT process within community contexts, including patients and relatives, care home staff members and primary care clinicians. Our study was limited by the under-representation of people from minority ethnic groups in the patient/relative data.^15^ Further research is needed to examine ReSPECT decision-making in emergency situations and include ambulance staff and other out-of-hours responders.

## Conclusion

The use of ReSPECT in community settings can facilitate best interests decision-making in an emergency by providing evidence of patient values and preferences regarding treatment and care. It is viewed positively by patients and relatives, GPs and specialist nurses and care home staff. However, as currently used, ReSPECT recommendations may not always reflect patient preferences, particularly when attending clinicians need to interpret recommendations that did not anticipate the current emergency. This may compromise best interests decision-making. Recording patients’ values and preferences in greater detail on ReSPECT plans, rather than focusing on specific recommendations, may help overcome this challenge, in providing attending clinicians with richer contextual information through which to interpret treatment recommendations.

## Data Availability

All data relevant to the study are included in the article or uploaded as supplementary information. Although the data in this qualitative study have been pseudonymised, it is possible that with access to raw data individuals might be identifiable. The data are not suitable for sharing beyond what is contained within the manuscript. Further information can be obtained from the corresponding authors.
