# Supplementary material for: Using the Recommended Summary Plan for Emergency Care and Treatment (ReSPECT) in a community setting: does it facilitate best interests decision-making?
Source: J Med Ethics. 2025 Jan 19;51(8):526–32. doi: 10.1136/jme-2024-110144 (PMC12322382; doi:10.1136/jme-2024-110144)
Supplement: Supplementary data [file jme-2024-110144supp001.pdf]

Eli K, Bernstein CJ, Harlock J, *et al*. Using the Recommended Summary Plan for Emergency Care and Treatment in a community setting: does it facilitate best interests decision-making?

Supplemental material: coding framework

Ethical concepts (categories)	Codes
1. Individual Autonomy	1) Control of ReSPECT initiation<br>2) Changing priorities during illness course<br>3) Patients' views not considered (in completion or in use of form)<br>4) Control of form/information<br>5) Patient understanding<br>6) Patient capacity<br>7) Empowers patient/reflects patient wishes<br>8) Advocating for one's own wishes<br>9) Clinician autonomy<br>10) Responsibilizing patient for others' wellbeing<br>11) Patient responsibility
2. Protecting patient from harm	1) Timing of conversation<br>2) Assumptions that form is DNACPR<br>3) Recommendations not being followed<br>4) Form protects patient from inappropriate treatment<br>5) Difficulty interpreting form<br>6) Availability of form for decision making<br>7) Form inadequately completed<br>8) Form prevents patient from receiving treatment<br>9) Caring for patient's emotional wellbeing<br>10) Multiple forms in circulation<br>11) Can't address all scenarios<br>12) Restricted conversation<br>13) Availability of form
3. Best interests	1) Person centred care<br>2) Responding to changes in health and care needs<br>3) Advocating for patient<br>4) Shared decision-making<br>5) Clinician responsibility to make a best interest decision
4. Trust	1) Who can have the conversation/fill in form<br>2) Challenge if HCP doesn't know patient<br>3) Continuity of care<br>4) Who has access to the information<br>5) Concern that recommendations will not be followed<br>6) Confidence that recommendations will be followed<br>7) General mistrust in HCPs<br>8) Good rapport<br>9) Nuanced/guided process<br>10) Importance of patient doctor relationship<br>11) Concern that other treatments will be limited

Eli K, Bernstein CJ, Harlock J, *et al*. Using the Recommended Summary Plan for Emergency Care and Treatment in a community setting: does it facilitate best interests decision-making?

5. Moral importance of family	1) Wanting to protect family<br>2) Involving family in conversation<br>3) Family's wishes/interests<br>4) Informing about decision<br>5) Caring for family's emotional wellbeing<br>6) Advocating for patient<br>7) facilitating understanding
6. Ethical importance of context	1) Impact of COVID<br>2) Cultural gaps between patient and clinicians<br>3) Impact of resources on deliverability of recommendations
7. Benefits to HSCPs	1) Guides HSCPs in acute situations (helps decision making)<br>2) Reassures that decision is in line with patient wishes<br>3) Form filling as good citizenship<br>4) Carers feeling disempowered<br>5) Carers feeling conflicted<br>6) Care homes bearing liability<br>7) Protects HSCPs from trauma<br>8) Broadens understanding of treatment options<br>9) Supports difficult conversations<br>10) Reduces conflict with families<br>11) Helps understand patients holistically<br>12) Having an important conversation<br>13) Feeling conflicted about following recommendations
8. Protecting the health care system	1) Reducing admissions<br>2) Strains staff resources
